# Patterns and sociodemographic influences on occupational physical activity at the Federal Rural University of Rio de Janeiro

**DOI:** 10.47626/1679-4435-2026-1523

**Published:** 2026-02-27

**Authors:** Rayssa Fernanda Garcia Nogueira Palau, Mariah Violla Carvalho, Marcello Silva Vellasco, Aldair José de Oliveira

**Affiliations:** 1 Universidade Federal Rural do Rio de Janeiro, Departamento de Educação Física e Desporto, Seropédica, RJ, Brazil

**Keywords:** health promotion, sociodemographic factors, occupational health, exercise., promoção da saúde, fatores sociodemográficos, saúde ocupacional, exercício físico.

## Abstract

**Introduction:**

Physical activity performed in the occupational context is related to both physical health and workers’ well-being. Understanding how sociodemographic factors influence this type of activity can support the development of strategies to promote health in the workplace.

**Objectives:**

To analyze the relationship between sociodemographic aspects and the practice of occupational physical activity among employees of the Federal Rural University of Rio de Janeiro.

**Methods:**

This is an observational, cross-sectional study conducted with employees of the Federal Rural University of Rio de Janeiro. Data were collected on age, sex, marital status, skin color, education level, and income, as well as on the frequency, intensity, and duration of occupational physical activity. Analyses included chi-square tests, analysis of variance, and Tukey’s post hoc test to compare groups.

**Results:**

Higher participation in occupational physical activities was observed among men, as well as a higher average time spent on such tasks among workers with lower education and income levels. Older employees showed longer exposure to occupational physical activities. Differences related to skin color were also identified, indicating potential structural inequalities in the workplace.

**Conclusions:**

The results show that sociodemographic factors significantly influence participation in and intensity of occupational physical activity. Identifying groups more exposed to high physical demands can guide institutional policies aimed at promoting health, preventing occupational diseases, and improving working conditions, with a focus on equity and inclusion.

## Resumo

**Introdução:**

A atividade física realizada no contexto ocupacional está relacionada tanto à saúde física quanto ao bem-estar dos trabalhadores. Compreender como fatores sociodemográficos influenciam esse tipo de atividade pode auxiliar na formulação de estratégias de promoção da saúde no ambiente de trabalho.

**Objetivos:**

Analisar a relação entre aspectos sociodemográficos e a prática de atividade física no contexto ocupacional entre servidores da Universidade Federal Rural do Rio de Janeiro.

**Métodos:**

Trata-se de um estudo observacional, de caráter transversal, realizado com servidores da Universidade Federal Rural do Rio de Janeiro. Foram coletadas informações sobre idade, sexo, estado civil, cor de pele, escolaridade e renda, bem como dados sobre frequência, intensidade e duração da atividade física ocupacional. As análises incluíram testes de qui-quadrado, análise de variância e teste *post hoc* de Tukey para a comparação entre grupos.

**Resultados:**

Observou-se maior participação masculina em atividades físicas ocupacionais, além de maior média de tempo dedicado a essas tarefas entre trabalhadores com menor escolaridade e renda. Servidores mais velhos apresentaram maior tempo de exposição a atividades físicas ocupacionais. Também foram identificadas diferenças associadas à cor de pele, indicando potenciais desigualdades estruturais no ambiente de trabalho.

**Conclusões:**

Os resultados demonstram que fatores sociodemográficos influenciam de forma significativa a participação e a intensidade da atividade física ocupacional. A identificação de grupos mais expostos a elevadas cargas físicas pode orientar políticas institucionais voltadas à promoção da saúde, prevenção de doenças ocupacionais e melhoria das condições de trabalho, com enfoque na equidade e inclusão.

## INTRODUCTION

Physical activity (PA) corresponds to any bodily movement produced by skeletal muscles that results in energy expenditure above resting levels, such as walking, dancing, and climbing stairs [1]. PA can be classified into different domains, such as leisure-time physical activity (LTPA) and occupational physical activity (OPA) [2]. OPA is performed in the workplace and includes activities such as standing for long periods and moving between sectors. Understanding OPA also requires an analysis of its historical evolution.

During the First Industrial Revolution, in the 18th and 19th centuries, the introduction of machines reduced the need for manual labor and imposed a more intense and physically demanding production pace. This process profoundly transformed the world of work and directly influenced OPA levels [3,4]. Later, with the Technological Revolution, from the late 20th century onward, many physical tasks began to be replaced by automated processes. Even so, sectors such as construction and logistics continue to require high physical effort, which, although it increases productivity, may pose health risks to workers [5,6].

The World Health Organization (WHO) recommends 150-300 minutes of moderate PA per week or 75-150 minutes of vigorous PA [7,8], without distinguishing between different activity domains. In the occupational context, moderate activities include brisk walking and carrying light objects, whereas vigorous activities involve, for example, lifting heavy loads and handling stock [2]. Although OPA may contribute to improved cardiovascular health and reduced chronic diseases [9], it may also be associated with adverse effects. High physical demands can lead to fatigue and compromise productivity [10]. In addition, studies indicate an association between high levels of OPA and poorer self-rated health, in contrast to the widely recognized benefits of LTPA [10,11].

In this context, the so-called physical activity paradox stands out, according to which the occupational domain may be associated with health impairment [12,13]. Maintaining static postures and continuously repeating movements increase the risk of injuries, which may counteract the cardiovascular benefits observed in LTPA [14]. Intense OPA is more frequent among individuals with lower income and education levels and in unhealthy work environments [15]. Moreover, sociodemographic variables, such as gender, age, marital status, and skin color, may influence the pattern of exposure to OPA [16,17].

With regard to gender, high-intensity occupational activities are traditionally more often performed by men [18,19]. One study indicated that men engaged in high-intensity OPA showed an 18% increase in mortality risk compared with those performing low-intensity occupational activities [20].

A prospective cohort study conducted in Norway with approximately 46,000 participants aged 18 to 65 years observed that younger workers tend to engage more frequently in vigorous-intensity OPA, whereas older workers are more likely to perform light-intensity tasks [21]. Regarding marital status, a cross-sectional study conducted in Salvador, Bahia, with 2,305 adults reported a higher proportion of OPA among married individuals, followed by those who self-identified as single [19].

With respect to skin color/race, non-White workers often occupy positions that require greater physical effort [16]. Regarding income and education, lower levels are usually associated with more precarious and socially devalued occupations, characterized by long working hours and activities of at least moderate intensity. This scenario may reduce the time available for other practices and negatively impact quality of life [16,22].

The relationship between OPA and the aforementioned sociodemographic variables constitutes a relevant topic for public health and workers’ quality of life. Understanding these interactions contributes to deepening knowledge about inequalities in the workplace, considering that certain population groups may be more exposed to high levels of occupational PA, which may have distinct repercussions for their health.

Therefore, considering the potential adverse effects of OPA on workers’ health and well-being, this study aims to analyze how sociodemographic factors, such as age, sex, marital status, skin color/race, education, and income, are associated with the weekly frequency, intensity, and duration of OPA among employees of Universidade Federal Rural do Rio de Janeiro (UFRRJ).

## METHODS

This study corresponds to the baseline analysis of the cohort Longitudinal Study of the Determinants of Physical Activity. Data collection was conducted after approval by the research ethics committee, through a self-administered questionnaire applied to 1,160 employees of a public university, of both sexes, aged between 23 and 75 years. For the present analysis, 11 participants who did not answer the questions related to OPA were excluded, resulting in a final sample of 1,149 employees.

The assessment of PA was carried out using the International Physical Activity Questionnaire, and, in this study, only the domain related to OPA was considered. The variables used in the analyses were weekly frequency, intensity (moderate and/or vigorous), and total time of OPA.

The sociodemographic variable “age group” was categorized as follows: “Young” (23-38 years), “Adult” (39-55 years), and “Older adult” (≥ 56 years). To construct the continuous variable referring to OPA time, the reported hours were converted into minutes. Subsequently, the minutes corresponding to vigorous activity were weighted and added to the minutes of moderate activity, resulting in the total OPA time.

Initially, a descriptive analysis of the variables was performed, including means, standard deviations, and minimum and maximum values. The chi-square test was used to assess associations between categorical variables. To compare the mean OPA time according to gender, Student’s *t* test was applied. Analysis of variance was used to compare the mean OPA time across categories of income, occupation, education, marital status, age group, and skin color/race. For this stage, groups that chose not to report certain variables were excluded. When significant differences were identified, Tukey’s post hoc test was applied to determine which groups differed from each other, adopting a significance level of p ≤ 0.05. Statistical analyses were performed using R software, version 4.3.3.

## RESULTS

The investigated sample shows a predominance of male individuals, married or in a stable union, and with a high level of education. The general sociodemographic characteristics are described in Table 1.

**Table 1:** General characteristics of the sample

Sociodemographic variables		n (%)	
Gender			
Female		513 (44.6%)	
Male		636 (55.4%)	
Income			
Up to R$ 6.212		386 (33.6%)	
From R$ 6.212 to R$ 10.212		319 (27.8%)	
More than R$ 10.213		417 (36.3%)	
Preferred not to report		27 (2.3%)	
Occupation			
Faculty		520 (45.3%)	
Administrative staff		628 (54.7%)	
Preferred not to report		1 (0.1%)	
Education			
Complete/incomplete elementary school		21 (1.8%)	
High school		102 (8.9%)	
Higher education		127 (11.1%)	
Postgraduate		873 (76%)	
Preferred not to report		26 (2.3%)	
Marital status			
Single		376 (32.7%)	
Married/stable union		726 (63.2%)	
Widowed		18 (1.6%)	
Preferred not to report		29 (2.5%)	
Age			
Yong adult		294 (25.6%)	
Adult		515 (44.8%)	
Older adult		263 (22.9%)	
Preferred not to report		77 (6.7%)	
Skin color/race			
Asian		10 (0.9%)	
White		622 (54.1%)	
Indigenous		3 (0.3%)	
Black		118 (10.3%)	
Brown		342 (29.8%)	
Preferred not to report		54 (4.7%)	
Moderate frequency			
0 day		927 (80.7%)	
1-3 days		146 (12.7%)	
4-7 days		76 (6.6%)	
Vigorous frequency			
0 day		1,037 (90.3%)	
1-3 days		74 (6.4%)	
4-7 days		38 (3.3%)	
Continuous variable	Mean±SD	Minimum	Maximum
Minutes of activity	46.05±144.6	0	1.440

SD: standard deviation.

The results indicated that, in the domain of moderate OPA, men reported a higher weekly frequency compared with women, although this difference did not reach statistical significance (p > 0.05). This same pattern was observed for the other sociodemographic variables analyzed. Details are presented in Table 2.

**Table 2:** Chi-square test analysis of the moderate physical activity frequency variable

	0 day	1-3 days	4-7 days	p-value
Gender				0.06
Female	429 (46.3%)	57 (39%)	27 (35.5%)	
Male	498 (53.7%)	89 (61%)	49 (64.5%)	
Income				0.15
Up to R$ 6.212	301 (32.5%)	54 (37%)	31 (41.8%)	
From R$ 6.212 to R$ 10.212	263 (28.4%)	37 (25.3%)	19 (25%)	
More than R$ 10.213	336 (36.2%)	55 (37.7%)	26 (34.2%)	
Preferred not to report	27 (2.9%)	0 (0%)	0 (0%)	
Occupation				0.18
Faculty	419 (45.2%)	75 (51.4%)	25 (34.2%)	
Administrative staff	507 (54.7%)	70 (48.6%)	50 (65.8%)	
Preferred not to report	1 (0.1%)	0 (0%)	0 (0%)	
Education				0.46
Complete/incomplete elementary school	307 (33.1%)	41 (28.1%)	28 (36.8%)	
High school	578 (62.4%)	102 (69.9%)	46 (60.5%)	
Higher education	15 (1.6%)	2 (1.4%)	1 (1.3%)	
Postgraduate	27 (2.9%)	1 (0.7%)	1 (1.3%)	
Preferred not to report				0.06
Marital status	15 (1.6%)	3 (2.1%)	3 (4%)	
Single	75 (8.1%)	16 (11%)	11 (14.5%)	
Married/stable union	107 (11.5%)	11 (7.5%)	9 (11.8%)	
Widowed	704 (75.9%)	116 (79.5%)	53 (69.7%)	
Preferred not to report	26 (2.8%)	0 (0%)	0 (0%)	
Age				0.14
Young adult	243 (26.2%)	28 (19.2%)	23 (30.3%)	
Adult	423 (45.6%)	66 (45.2%)	26 (34.2%)	
Older adult	204 (22%)	40 (27.4%)	19 (25%)	
Preferred not to report	57 (6.1%)	12 (8.2%)	8 (10.5%)	
Skin color				0.51
Asian	6 (0.6%)	4 (2.7%)	0 (0%)	
White	501 (54%)	80 (54.8%)	41 (53.9%)	
Indigenous	3 (0.3%)	0 (0%)	0 (0%)	
Black	94 (10.1%)	17 (11.6%)	7 (9.2%)	
Brown	279 (30.10%)	38 (26%)	25 (32.9%)	
Preferred not to report	44 (4.7%)	7 (4.8%)	3 (3.9%)	

In vigorous OPA, gender showed a statistically significant association (p ≤ 0.05), as did education level, suggesting a relationship between educational attainment and the frequency of this practice. The other sociodemographic variables did not demonstrate a significant association. Detailed results are presented in Table 3.

**Table 3:** Chi-square test analysis of the vigorous physical activity frequency variable

	0 day	1-3 days	4-7 days	p-value
Gender				0.0003^*^
Female	483 (46.6%)	20 (27%)	10 (26.3%)	
Male	554 (53.4%)	54 (73%)	28 (73.7%)	
Income				0.15
Up to R$ 6.212	339 (32.7%)	28 (37.8%)	19 (50%)	
From R$ 6.212 to R$ 10.212	295 (28.4%)	16 (21.6%)	8 (21.1%)	
More than R$ 10.213	376 (36.3%)	30 (40.5%)	11 (28.9%)	
Preferred not to report	27 (2.6%)	0 (%)	0 (%)	
Occupation				0.29
Faculty	472 (45.5%)	37 (50%)	11(28.9%)	
Administrative staff	564 (54.4%)	37 (50%)	27(71.1%)	
Preferred not to report	1 (0.1%)	0 (0%)	0(0%)	
Education				0.72
Complete/incomplete elementary school	344 (33.2%)	21 (28.4%)	11 (28.9%)	
High school	648 (62.5%)	51 (68.9%)	27 (71.1%)	
Higher education	17 (1.6%)	1 (1.4%)	0 (0%)	
Postgraduate	28 (2.7%)	1 (1.4%)	0 (0%)	
Preferred not to report				0.0003^*^
Marital status	16 (1.5%)	2 (2.7%)	3 (7.9%)	
Single	89 (8.6%)	4 (5.4%)	9 (23.7%)	
Married/stable union	122 (11.8%)	5 (6.8%)	0 (0%)	
Widowed	784 (75.6%)	63 (85.1%)	26 (68.4%)	
Preferred not to report	26 (2.5%)	0 (0%)	0 (0%)	
Age				0.17
Young adult	269 (25.9%)	16 (21.6%)	9 (23.7%)	
Adult	473 (45.6%)	31 (41.9%)	11 (28.9%)	
Older adult	227 (21.9%)	22 (29.7%)	14 (36.8%)	
Preferred not to report	68 (6.6%)	5 (6.8%)	4 (10.5%)	
Skin color/race				0.50
Asian	9 (0.9%)	1 (1.4%)	0 (0%)	
White	569 (54.9%)	37 (50%)	16 (42.1%)	
Indigenous	3 (0.3%)	0 (0%)	0 (0%)	
Black	102 (9.8%)	12 (16.2%)	4 (10.5%)	
Brown	306 (29.5%)	19 (25.7%)	17 (44.7%)	
Preferred not to report	48 (4.6%)	1 (2.6%)	1 (2.6%)	

* values marked with an asterisk indicate that the p-value is less than 0.05.

Data regarding OPA time showed statistically significant differences (p ≤ 0.05) among some sociodemographic variables. Women presented a mean of 32±111.6 minutes, whereas men recorded 57.3±165.8 minutes (p = 0.002). Regarding income, individuals with lower income levels showed a mean of 63.80±178.3 minutes, compared with 34.57±115.54 minutes at the intermediate level (p = 0.02).

Marital status did not show a significant association (p ≥ 0.05), although married individuals or those in a stable union recorded a higher mean (50.6±156.8 minutes). Similarly, no significant differences were observed according to occupation, although administrative staff presented a mean of 52.7±164.6 minutes, whereas faculty recorded 38±115.8 minutes.

Regarding education, a statistically significant difference was observed (p = 0.0001), with emphasis on comparisons between elementary school and higher education (p = 0.002) and between elementary school and postgraduate education (p = 0.006). The mean OPA time was 146.19±270 minutes for elementary school, 92.84±238.5 for high school, 25±78.68 for higher education, and 42.52±133.31 minutes for postgraduate education.

The age variable also showed a significant association (p = 0.001), with a higher mean observed among older adults (68.27±175.113 minutes) compared with adults (p = 0.006).

Regarding skin color/race, no significant differences were identified (p ≥ 0.05). The highest means were recorded among Black individuals (57.712±174.103 minutes), followed by Asian individuals (54±150.864 minutes) and Brown individuals (53.87±161.14 minutes). Detailed information is presented in Table 4.

**Table 4:** Analysis of variance of occupational physical activity time

	Mean±SD	p-value
Gender		0.002^*^
Female	32±111.62	
Male	57±165.89	
Income		0.01^*^
Up to R$ 6.212	63.80±178.3	
From R$ 6.212 to R$ 10.212	34.57±115.54	
More than R$ 10.213	41.36±132.66	
Occupation		0.07
Faculty	38.06±115.8	
Administrative staff	52.7±164.69	
Education		0.0001^*^
Complete/incomplete elementary school	146.19±270.06	
High school	92.84±238.52	
Higher education	25.59±78.68	
Postgraduate	42.52±133.31	
Marital status		0.52
Single	40.41±125.34	
Married/stable union	50.61±156.81	
Widowed	37.22±98.44	
Age		0.001^*^
Young adult	41.05±146.95	
Adult	32.73±107.06	
Older adult	68.27±175.13	
Skin color		0.37
Asian	54±150.86	
White	40.33±132.22	
Indigenous	0±0	
Black	57.71±174.10	
Brown	53.87±161.14	

SD: standard deviation.

p-value derived from Student’s t test, analysis of variance, and Tukey’s post hoc test.

* values marked with an asterisk indicate p ≤ 0.05.

## DISCUSSION

This study analyzed the association between sociodemographic characteristics and the frequency, intensity, and duration of OPA among employees of UFRRJ, showing that variables such as gender, income, age, education, and occupation influence the level of exposure to this type of activity.

Men showed greater participation in OPA, regardless of intensity and time spent, a result consistent with findings from other studies [19]. Cultural issues and social constructions of gender, which have historically associated manual labor with the male sphere, may explain part of this difference. Sectors traditionally occupied by men, such as construction and heavy industry, contribute to maintaining this pattern. In addition, male socialization often encourages the performance of activities that demand greater physical effort, whereas women tend to be directed toward functions related to care and domestic tasks, although these may also involve significant physical effort.

Employees with lower income presented higher means and frequencies of OPA, a result similar to that described in the literature [15,23]. Older adults, in turn, showed higher mean time and weekly frequency of OPA, differing from the study by Dalene et al. [21], which identified greater occupational engagement in physical activities among younger individuals. In this context, interventions aimed at LTPA for this group may contribute positively to health and productivity [12]. Lower education was also associated with higher levels of OPA, as observed by Oliveira et al. [15]. Regarding occupation, administrative staff presented higher means and frequencies compared with faculty, with these three factors possibly connected due to lower salaries and functions with greater physical demands, in addition to entry requirements that often involve lower educational levels, which tends to concentrate certain sociodemographic characteristics in this group.

With regard to marital status, married employees or those in a stable union presented the highest mean OPA time. However, when weekly frequency was analyzed, widowed individuals showed greater involvement in both moderateand vigorous-intensity activities. A previous study [19] partially supports this observation by indicating greater participation in OPA among individuals who are married or in a stable union. These findings suggest that marital status may influence the amount of time dedicated to PA in the occupational environment.

Regarding skin color/race, non-White individuals presented higher mean OPA time, possibly because they occupy roles that demand greater physical effort and require lower levels of education. Considering that entry into public service occurs through competitive examinations, selection tends to favor candidates with higher educational attainment. The implementation of quota policies, which reserve part of the positions for Black, Brown, and Indigenous individuals, constitutes an important strategy to reduce historical inequalities. Even so, it is possible that many non-White employees did not enter through this policy, which may contribute to their greater concentration in positions that require lower formal qualifications and greater physical effort. In this scenario, time dedicated to OPA above baseline levels may be more frequent among these groups.

The internal heterogeneity among groups and the influence of structural racism in the workplace, including processes of occupational devaluation and experiences of discrimination [24], reinforce the importance of considering socioeconomic and cultural factors in the analysis of OPA. These disparities highlight the need for institutional policies aimed at promoting equity and protecting workers’ health.

## CONCLUSIONS

The present study highlighted relevant aspects of OPA among employees of UFRRJ, demonstrating that sociodemographic characteristics directly influence both participation in and the intensity of this practice. The results indicate that men tend to be more involved in OPA than women, reinforcing the need to reflect on the persistence of gender stereotypes in the workplace. Likewise, workers with lower income and education presented higher mean levels of OPA, highlighting socioeconomic inequalities related to the type of occupation and to physical work demands.

The analysis by age group showed that older workers remain engaged for longer periods in physically demanding activities, which contrasts with common perceptions about aging in the workplace and highlights the importance of policies aimed at the well-being of this population. The skin color/race variable also raises relevant considerations by suggesting possible influences of structural inequalities and subtle discrimination in the distribution of tasks and occupational experiences, indicating the need for further in-depth investigations.

This study contributes to expanding the understanding of how social and economic structures shape the experience of OPA and reinforces that occupational health interventions should consider the specificities of different groups. Understanding how physical demands fall unevenly on certain workers may assist in identifying situations in which sociodemographic variables overlap, intensifying work overload and its impacts on performance and well-being.

The relevance of preventive actions in the workplace should also be highlighted, such as the implementation of active breaks, guided stretching, and other strategies that may reduce pain, fatigue, and adverse effects resulting from OPA. Measures associated with the organization and adjustment of occupational tasks tend to contribute to the promotion of workers’ physical and mental well-being.

Finally, the findings presented provide important support for the formulation and improvement of occupational health policies. The identification of groups more exposed to high or inadequate physical workloads makes it possible to develop more targeted interventions aimed at preventing occupational diseases and promoting a healthier work environment for UFRRJ employees.
